# Somatic Genetic Predictive Testing for Therapeutic Decision-making in Women at Early Stage of Breast Cancer: What is The Role of Geneticists in Psychological Support?

**DOI:** 10.1192/j.eurpsy.2025.1773

**Published:** 2025-08-26

**Authors:** N. Bouayed Abdelmoula, B. Abdelmoula

**Affiliations:** 1 Genomics of Signalopathies at the Service of Precision Medicine LR23ES07, Medical University of Sfax, Sfax, Tunisia

## Abstract

**Introduction:**

**Somatic genomic variation testing has become an integral part of breast cancer management. Genetic tests based on gene expression profiles in cancer cells (biopsy or surgical specimen) are used for diagnostic, prognostic and theranostic purposes, helping to make therapeutic decisions: selection of adjuvant therapy, prediction of therapeutic responses and of breast cancer recurrence, in patients with early-stage breast cancer. The currently used gene expression assays including MammaPrint, Oncotype DX, Prosigna, etc..**

**Objectives:**

The aim of this study was to collect, through a recent review of the literature, the guidelines concerning psychological support before and after predictive genomic exploration in order to situate the role of the geneticist in this mental care.

**Methods:**

We conducted a comprehensive review of the scientific literature with the following keywords: genomic predictive diagnosis, breast cancer and mental health. Through the papers emerging from this research, we assessed guidelines concerning mental health care and genetic counselling.

**Results:**

There is not any guidelines or frameworks concerning the psycho-management of the new predictive genomic tests of breast cancer. Our review show that psychological support is systematically offered in oncology (psycho-oncology) and that the American Society of Clinical Oncology (ASCO) has established guidelines for the management of anxiety and depression in adult survivors of cancer. Moreover, the most recent ASCO guidelines recommend the use of each genomic assay according to the level of evidence available for specific clinical conditions, in breast cancer. There are few papers dealing with genetic counselling and psychological support during the oncotype Dx and MammaPrint tests that are the incorporated into in everyday practice and recommended in clinical guidelines for women with node-negative, hormone-receptor-positive breast cancer. While, in pre-test genetic counselling is well established in breast cancer genetic predisposition, the frameworks in predictive tests and their emotional and psychological impact during prescription, testing and discussion of results as well as the therapeutic choices, are not clear until now.

**Conclusions:**

Genetic counseling includes both informative and educational aspects, as well as advice on personal decisions that may affect the rest of the patient’s life. During next-generation predictive testing, the objectives would also be to provide the patient with the ability to gain a sense of control by reducing uncertainty, to determine her own level of risk of recurrence and her therapeutic chances and to receive psychological support during the shared therapeutic decision. Thus, the role of the genetic counsellors must be an essential key in the recent era of mainstreaming of genomic sequencing.

**Disclosure of Interest:**

None Declared

